# Oncological and prognostic impact of lymphovascular invasion in Colorectal Cancer patients

**DOI:** 10.7150/ijms.53555

**Published:** 2021-02-10

**Authors:** Xiaofei Wang, Yinghao Cao, Miaomiao Ding, Junqi Liu, Xiaoxiao Zuo, Hongfei Li, Ruitai Fan

**Affiliations:** 1 Department of radiotherapy, The First Affiliated Hospital of Zhengzhou University, Zhengzhou, Henan, China.; 2 Department of Gastrointestinal Surgery, Union Hospital, Tongji Medical College, Huazhong University of Science and Technology, Wuhan, Hubei, China.; 3 Department of Ultrasonography, The First Affiliated Hospital of Zhengzhou University, Zhengzhou, Henan, China.

**Keywords:** colorectal cancer, lymphovascular invasion, surgical resection, TCGA database, survival nomogram

## Abstract

**Objectives:** Lymphovascular invasion (LVI) is correlated with unfavorable prognoses in several types of cancers. We aimed to identify the informative features associated with LVI and to determine its prognostic value in colorectal cancer (CRC) patients.

**Methods:** We retrospectively analyzed 1,474 CRC patients admitted in Wuhan Union Hospital between 2013 and 2017 as the development cohort and 549 CRC patients from The Cancer Genome Atlas (TCGA) database as the validation cohort. Logistical and Cox regression analyses were conducted to determine the oncological and prognostic significance of LVI in CRC patients. A survival nomogram based on LVI status was established using the Wuhan Union cohort and validated using TCGA cohort.

**Results:** The LVI detection rates were 21.64% in the Wuhan Union cohort and 35.15% in TCGA cohort. LVI was closely correlated with advanced T stage, N stage, and TNM stage. LVI positivity was an independent biomarker for unfavorable overall survival (hazard ratio [HR]=2.25, 95% confidence interval [CI]=1.70-2.96, P<0.0001) and worse disease-free survival (HR=2.34, 95% CI=1.76-3.12, P<0.0001) in CRC patients. The survival nomogram incorporating LVI exhibited good predictive performance and reliability in the Wuhan Union cohort and TCGA cohort.

**Conclusion:** LVI is a significant indicator of advanced stage and is remarkably correlated with worse prognosis in CRC patients. The survival nomogram incorporating LVI may assist clinicians to better strategize the therapeutic options for patients with CRC.

## Introduction

Colorectal cancer (CRC) is one of the most common types of malignant tumors occurring in the gastrointestinal tract and the main cause of cancer-related death 1-3. Despite the great advances in surgical and targeted therapies 4, the long-term survival of CRC patients with metastasis is far from our expectation 5. A specific pathological variable associated with CRC metastasis is lymphovascular invasion (LVI) 6. LVI is defined as the presence of tumor cells within the lymphatic or vascular channels 7, which is a common histopathological finding in CRC. LVI is an early sign of lymph node metastasis and increases the risk for micrometastasis in patients with localized CRC 8. Hence, clarifying the oncological impact and prognostic significance of LVI is of great significance for patients with CRC 9, 10.

Although a panel of clinical studies have explored the association between LVI and survival outcomes in patients with CRC, the results were inconsistent 7, 11, 12. No study has established a survival nomogram based on LVI for accurate stratification of CRC patients with high risk for poor outcomes. More importantly, the clinical features closely related to the incidence of LVI are still unknown. In our previous study 13, we investigated the prognostic value of another ominous pathologic feature, perineural invasion, of CRC. In this study, we aimed to identify the critical clinical characteristics associated with LVI and to create a survival nomogram based on LVI status for patients with CRC.

## Methods

### Study population from Wuhan Union cohort

Newly diagnosed patients with confirmed CRC admitted in Wuhan Union Hospital between July 2013 and September 2017 were enrolled in this study 13. Data on demographic characteristics, tumor markers, staging, pathology, treatment, and survival outcomes (overall survival [OS] and disease-free survival [DFS]) were retrospectively collected from each patient. As for follow-up, the frequency was twice a half year after surgical resection, and then the frequency was twice a year. OS refers to the period from the date of surgical resection to the time of death by any cause. DFS refers to the period from the time of surgical removal to the date of recurrence or death by CRC. Staging was implemented based on the 8^th^ edition of the American Joint Committee on Cancer TNM staging system. Patients with histologically diagnosed CRC, patients whose LVI was diagnosed based on a postoperative histopathology report, and CRC patients who underwent surgical treatment were included in the study. By contrast, patients whose CRC was complicated with other malignant tumors and CRC patients with missing critical information were excluded. Moreover, children patients and patients without surgical resection were also excluded from this study. A total of 1,474 CRC patients with intact data were included in this study. Informed consent was obtained from all participants prior to the commencement of the study. The study was reviewed and approved by the ethics Committee of Wuhan Union Hospital (no. 2018-S377).

### CRC patients from The Cancer Genome Atlas (TCGA) database

For independent validation, TCGA database 14 was searched to screen CRC patients who fulfilled the inclusion criteria. Over 549 CRC patients with complete clinical information and follow-up data were identified from the database. A total of 1,474 CRC patients from Wuhan Union Hospital were utilized as the development cohort (Wuhan Union cohort), while 549 CRC patients from TCGA database were used as the validation cohort (TCGA cohort).

### Development of the survival nomogram

The clinical features, which were significantly correlated (P<0.05) with the OS of CRC patients, were identified by conducting a multivariable Cox analysis in the development cohort. These characteristics were selected for the construction of an OS nomogram. The predictive discrimination of the OS nomogram was determined using the receiver operating characteristic (ROC) analysis. The calibration ability of the OS nomogram, as reflected by goodness of fit, was measured using a calibration curve. A DFS nomogram was also established in the same manner.

### Statistical analysis

All statistical analyses were performed using the SPSS 21.0 and R software (version 3.1.1). The chi-square test was employed to analyze the differences in categorical indexes between LVI+ and LVI- patients. Multivariable logistic regression was utilized to identify the independent variables affecting LVI+. Cox regression analysis was adopted to identify the potent prognostic factors in patients with CRC. ROC analysis was performed to assess the predictive ability of the survival nomogram. The Kaplan-Meier method and log-rank test were used to estimate the prognostic significance of LVI and the nomogram. Two-sided P values of less than 0.05 were considered significant.

## Results

### Features of the LVI+ and LVI- groups

LVI tumors were identified in 319 (21.64%) of the 1,474 CRC patients with CRC in the Wuhan Union cohort. As listed in Table 1, CRC patients with LVI+ exhibited more advanced T stage (P<0.001), N stage (P<0.001), and TNM stage (P<0.001) than those with LVI-. Among 549 CRC patients in TCGA cohort, 193 patients (35.15%) showed LVI+. Similar to the Wuhan Union cohort, CRC patients with LVI+ showed more advanced T stage (P=0.003), N stage (P<0.001), and TNM stage (P<0.001) than those with LVI-. Hence, the presence of LVI was closely correlated with aggressive tumor behavior in patients with CRC.

### Factors that independently affected the incidence of LVI

To determine which clinical variables could independently affect the incidence of LVI, univariable and multivariable logistical regression analyses were performed in the Wuhan Union cohort (Table 2). The multivariable logistical regression analysis showed that T4 stage (P=0.017), N3 stage (P<0.001), stage IV (P<0.001), and absence of radiotherapy (P=0.015) were independent variables affecting the incidence of LVI in patients with CRC.

### Prognostic value of LVI

**Figure 1:**
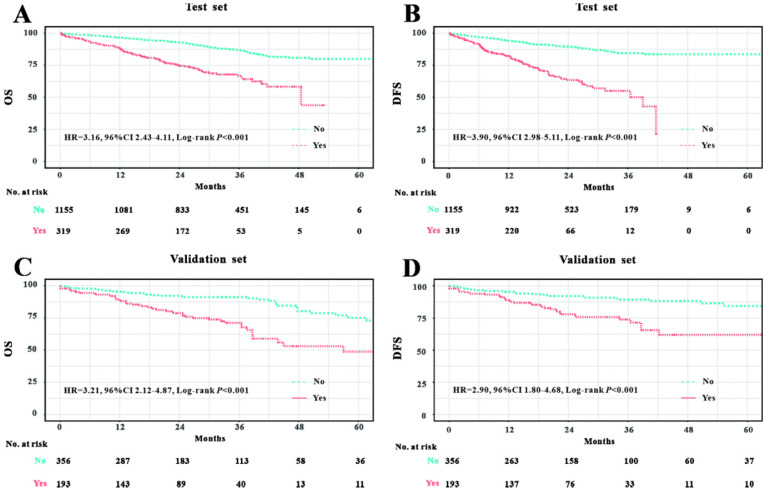
Prognostic significance of LVI in Wuhan Union cohort and TCGA cohort. LVI positivity was associated with unfavorable OS in CRC patients in Wuhan Union cohort (**A**) and TCGA cohort (**C**). LVI positivity was closely correlated with poor DFS in CRC patients in Wuhan Union cohort (**B**) and TCGA cohort (**D**).

We initially analyzed the prognostic value of LVI in the Wuhan Union cohort. As shown in Table 1, the LVI+ group possessed relatively higher death rate than the LVI- group (29.2% vs. 12.7%, P<0.001). Similarly, the LVI+ group had a higher rate of recurrence compared with the LVI- group (30.7% vs. 10.7%, P<0.001). We further adopted the Kaplan-Meier plots to estimate the prognostic significance of LVI and found that the LVI+ group exhibited worse OS (hazard ratio [HR]=3.16, 95% confidence interval [CI]=2.43-4.11, P<0.001, Figure 1A) and poorer DFS (HR=3.90, 95% CI=2.98-5.11, P<0.001, Figure 1C) than the LVI- group. In TCGA cohort, the LVI+ group demonstrated relatively higher mortality than the LVI- group (27.5% vs. 11.1%, P<0.001). Similarly, the LVI+ group showed higher rate of CRC recurrence than the LVI- group (20.7% vs. 8.4%, P<0.001). We further exploited Kaplan-Meier curves to determine the prognostic role of LVI in CRC patients from TCGA database; we found that the LVI+ group exhibited worse OS (HR=3.21, 95% CI=2.12-4.87, P<0.001, Figure 1B) and poorer DFS (HR=2.90, 95% CI=1.80-4.68, P<0.001, Figure 1D) than the LVI- group.

### Establishment of the survival nomogram

**Figure 2:**
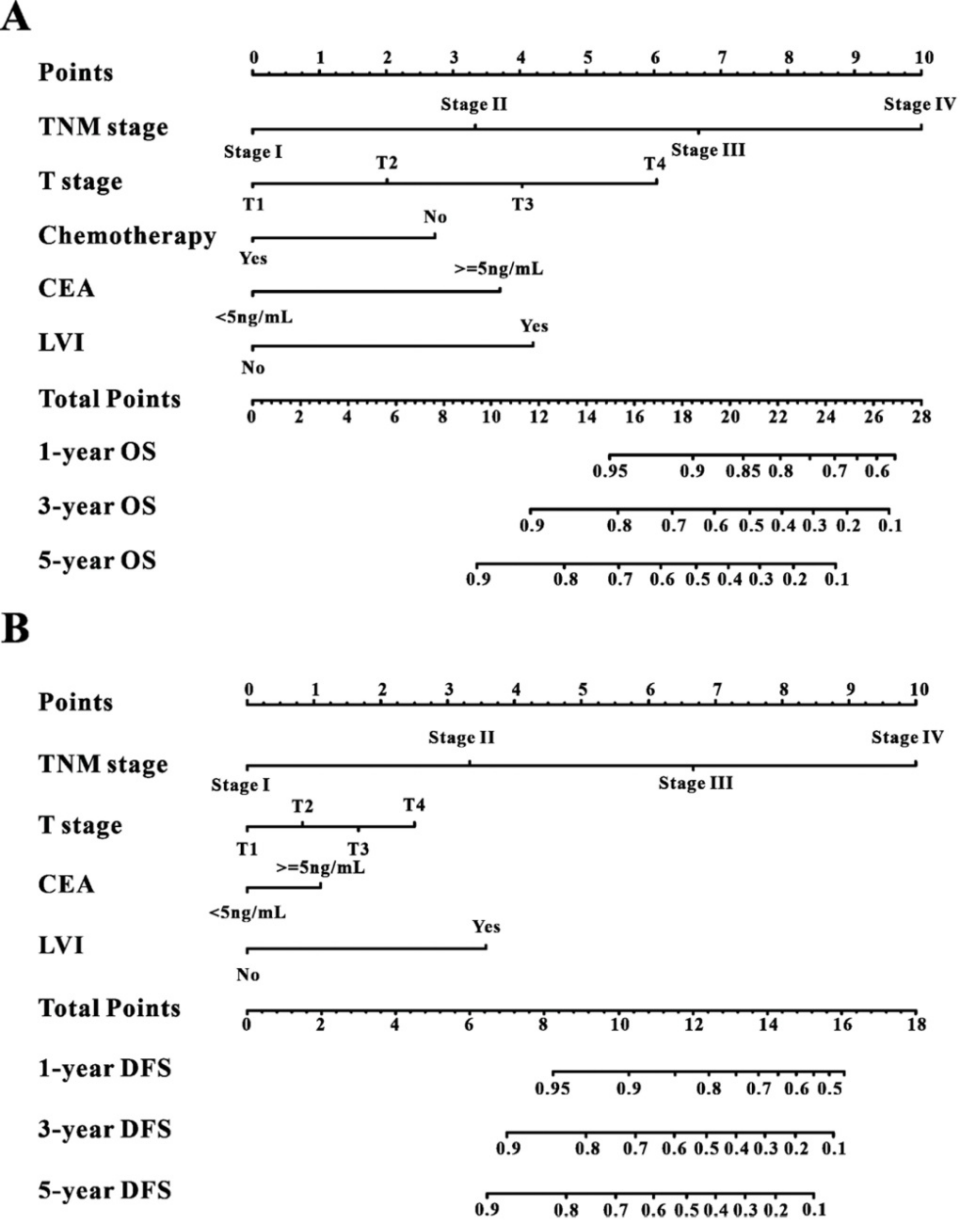
Construction of the survival nomogram in Wuhan Union cohort. The survival nomogram was applied for the prediction of the 1-year, 3-year and 5-year OS (**A**) and DFS (**B**) in CRC patients.

**Figure 3:**
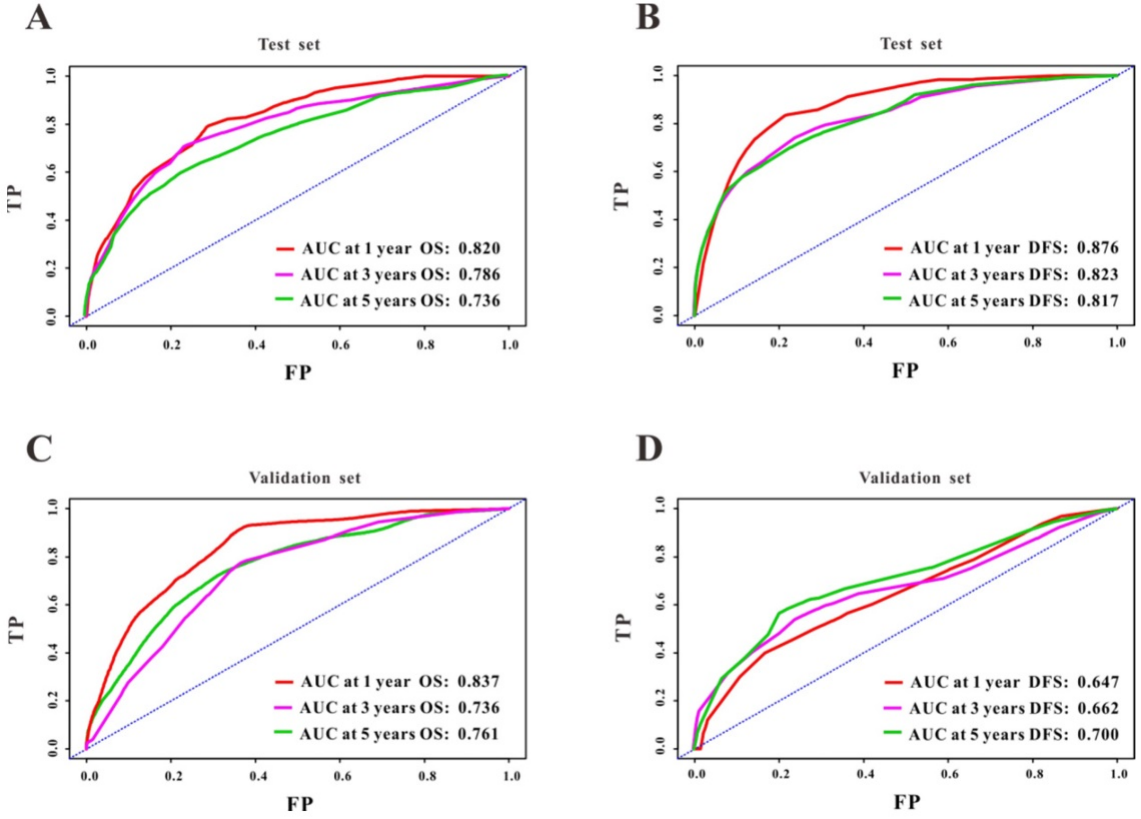
ROC curves of the survival nomogram for the prediction of OS and DFS. The survival nomogram exhibited good predictive performance for OS in Wuhan Union cohort (**A**) and TCGA cohort (**C**), and DFS in CRC patients in Wuhan Union cohort (**B**) and TCGA cohort (**D**).

**Figure 4:**
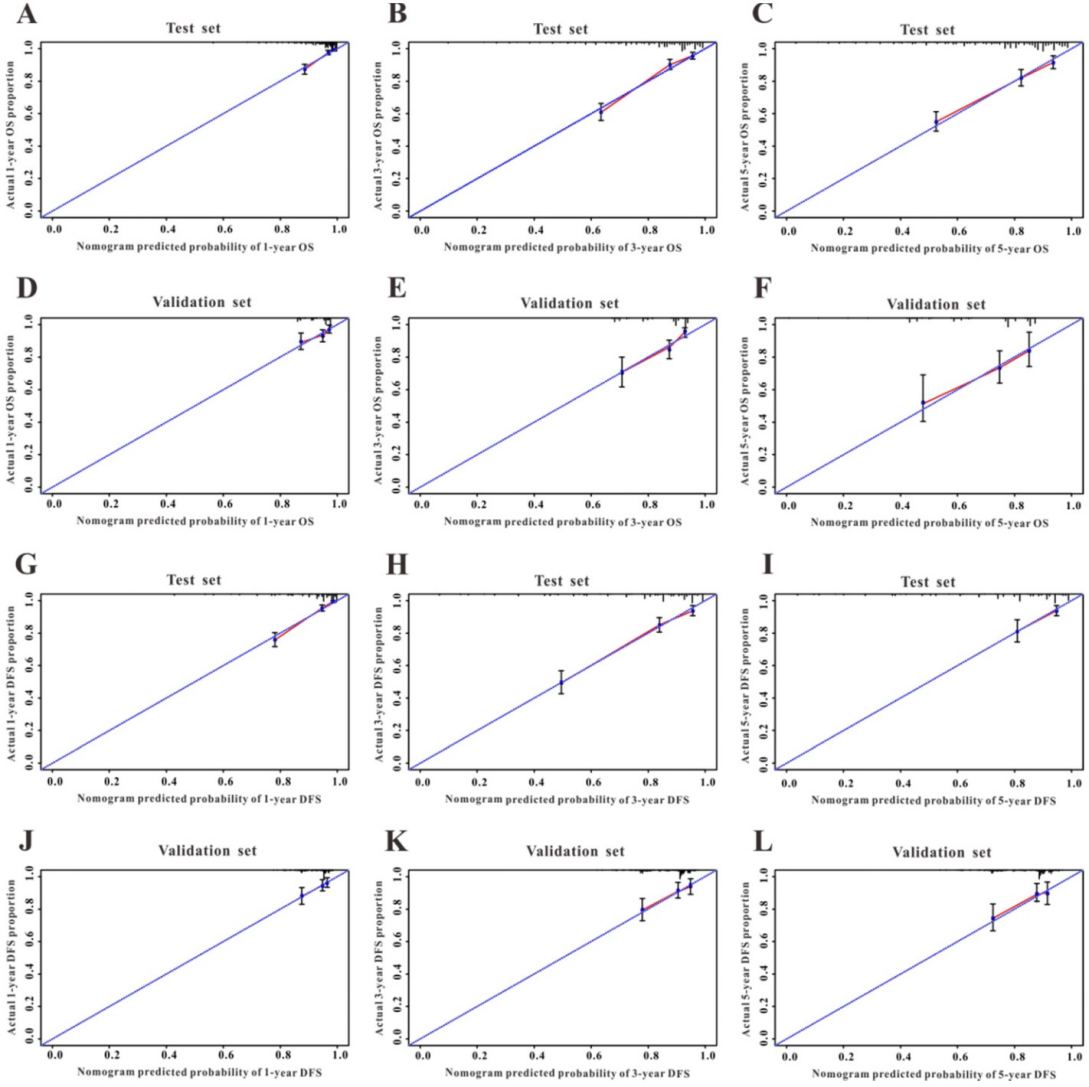
Calibration curves of the survival nomogram. Good agreement of the calibration plots for OS in Wuhan Union cohort (**A-C**) and TCGA cohort (**D-F**), and for DFS in CRC patients in Wuhan Union cohort (**G-I**) and TCGA cohort (**J-L**).

A Cox regression model was employed to explore the influence of LVI and other covariates on OS in patients with CRC from Wuhan Union cohort. As shown in Table 3, LVI (P<0.001), T4 stage (P=0.031), stage IV (P=0.016), absence of adjuvant chemotherapy (P<0.001), and carcinoembryonic antigen (CEA) (P<0.001) were all independent risk factors for unfavorable OS in patients with CRC. As shown in Figure 2A, the OS nomogram included five risk factors that may increase the probability of having a poor OS. The evaluative indexes, such as predictive performance measured using the AUC (1 year, 3 years, and 5 years: 0.82, 0.786, and 0.736, respectively), (Figure 3A) and calibration curves showed good agreement (Figure 4A-C). With regard to the DFS (Table 3), multivariable Cox analysis identified four critical variables that were significantly correlated with DFS, including LVI (P<0.001), T stage (P=0.021), TNM stage (P<0.001), and CEA (P=0.005). We also adopted the four critical indexes in order to develop a DFS nomogram for CRC patients (Figure 2B). The 1-year, 3-year, and 5-year AUC values of td-ROC were 0.876, 0.823, and 0.817, respectively (Figure 3C). In addition, the calibration curves showed that the 1-year, 3-year, and 5-year OS rates between the predicted DFS nomogram and actual observed values exhibited good concordance (Figure 4D-F).

### Validation of the survival nomogram in TCGA cohort

First, we used TCGA cohort to externally verify the discrimination and calibration of the OS nomogram. As shown in Figure 3B, the accuracy of the OS nomogram indicated by the AUC values (1 year, 3 years, and 5 years: 0.837, 0.736, and 0.761, respectively) was good. The calibration curves (Figure 4G-I) for 1 year, 3 years, and 5 years demonstrated the excellent calibration ability of the OS nomogram. In addition, the DFS nomogram showed the AUC values of 0.647, 0.662, and 0.761 for 1-year, 3-year, and 5-year recurrence among CRC patients (Figure 3D). The calibration curves (Figure 4J-L) displayed an excellent agreement in TCGA cohort for 1-year, 3-year, and 5-year DFS.

## Discussion

In this study, we initially investigated the oncological significance of LVI in CRC patients who underwent surgical resection and found that LVI was a histologic index of advanced stage in CRC. T4 stage, N3 stage, stage IV, and absence of radiotherapy were independent risk factors for LVI, which were never reported in the previous studies. Then, we further probed the prognostic value of LVI both in the Wuhan Union cohort and TCGA cohort, and the results were almost consistent. Finally, we constructed a survival nomogram based on the LVI status for the risk stratification of CRC patients who underwent surgical treatment in the Wuhan Union cohort. We also validated the survival nomogram using the clinical data from TCGA cohort. Indeed, this multicenter retrospective analysis systemically illustrated the oncological impact of LVI and was the first to establish a survival nomogram incorporating the LVI status for the risk stratification of patients with CRC.

The incidence of LVI was not unified in the published studies. Kim et al. 15 demonstrated an LVI incidence rate of 8.5% in patients with stage I CRC. A clinical study based on the Swedish colorectal cancer registry 11 reported an LVI incidence rate of 15% in patients with stage II CRC. A recent study from Germany showed an LVI detection rate of 22.0% in patients with stage II CRC. A clinical study conducted in 3,707 stage I-III CRC patients from South Korea reported an LVI incidence of 39.7%, which was highest among the rates reported in previous studies related to CRC. Another retrospective study 12 revealed that the overall detection rate of LVI was 12.3% among patients with CRC. A previous study 7 examining the National Cancer Data Base reported an LVI detection rate of 26.3%. In our study, the LVI detection rates were 21.64% in the Wuhan Union cohort and 35.15% in TCGA cohort, which was in line with the results of other studies related to LVI. More importantly, no study has determined the independent risk factors for LVI. The multivariable logistical regression proved that T4 stage, N stage, stage IV, and absence of radiotherapy were closely correlated with the occurrence of LVI, which can serve as a reference for predicting the risk of LVI in CRC patients who underwent surgery.

Although several studies have already reported the association between the presence of LVI and CRC, most of them investigated this association in patients with a specific stage, such as stage I 15, stage II 16 11, and stage I-III 17. Unfortunately, no study has evaluated the correlation of LVI with oncological and prognostic implications in CRC patients who underwent surgical resection. Our study included CRC patients in stage I-IV treated with surgical resection, and our results were more representative than those of previous studies. More importantly, we appropriately utilized TCGA database to validate the results of the Wuhan Union cohort and obtained similar results, implicating that our survival nomogram can be applicable to different populations. From this standpoint, our study could be viewed as a multicenter study with sufficient CRC patients.

The College of American Pathologists recommended the assessment of CRC patients for presence of LVI due to its highly important clinical significance 18. However, the clinical significance of LVI was somewhat underestimated by some studies. A great number of survival nomograms were created for the risk stratification of CRC patients with unfavorable outcomes 19-25. However, none of them included the significant features of LVI. In our study, a multivariable Cox regression was used to identify the clinical indexes that were significantly correlated with survival in CRC patients. Unsurprisingly, LVI positivity was a potent prognostic marker for both poor OS and DFS, and this finding was similar to the survival analysis results of other clinical studies. Encouragingly, the survival nomogram incorporating LVI obtained more excellent predictive performance in the prediction of survival than the TNM stage. Furthermore, when validated in TCGA cohort, the survival nomogram still possessed an extremely good predictive performance.

Although this is a multicenter research with relatively large sample size, two limitations still exist. One of the limitations was the retrospective nature of the study, and several relevant variables, such as tumor budding and microsatellite instability, were not included in our analysis. Another limitation was that the survival nomogram for CRC patients was not validated in the perspective cohort. Hence, well-designed perspective studies investigating the oncological and prognostic significance of LVI in CRC patients are warranted in the future.

## Conclusion

Our data showed that LVI may serve as a significant indicator for aggressive tumor behavior and is remarkably correlated with worse prognosis in CRC patients. Moreover, the survival nomogram containing LVI obtained an extremely good predictive ability for predicting OS and DFS in CRC patients, indicating that the survival nomogram can be utilized as a useful prognostic system for individual estimation of prognosis.

## Ethics Committee Approval and Patient Consent

The study plan was checked and approved prior to the beginning of the research by the ethics Committee of Wuhan Union Hospital (No. 2018-S377). The informed consent was obtained from the all the participants.

## Data Sharing Statement

All data in our study are available from the corresponding author upon reasonable request.

## Figures and Tables

**Table 1:** Clinicopathological characteristics of CRC patients in Wuhan Union and TCGA cohorts

Characteristics	Wuhan Union cohort (n=1474)			TCGA cohort (n=549)		
	LVI (n=319)	Non-LVI (1155)	*P* value	LVI (n=193)	Non-LVI (n=356)	*P* value
**Age, n (%)**			0.057			0.385
≥60 years	165 (51.7%)	528 (45.7%)		147 (76.2%)	259 (72.8%)	
<60 years	154 (48.3%)	627 (54.3%)		46 (23.8%)	97 (27.2%)	
**Gender, n (%)**			0.761			0.168
Male	190 (59.6%)	677 (58.6%)		90 (46.6%)	188 (52.8%)	
Female	129 (40.4%)	478 (41.4%)		103 (53.4%)	168 (47.2%)	
**Race, n (%)**			-			0.055
White	0 (0.0)	0 (0.0)		73 (37.8%)	160 (44.9%)	
Black	0 (0.0)	0 (0.0)		12 (6.2%)	30 (8.4%)	
Others	319 (100.0%)	1155 (100.0%)		108 (56.0%)	166 (46.6%)	
**Primary site, n (%)**			0.151			0.231
Left colon	105 (32.9%)	346 (30.0%)		82 (42.5%)	166 (46.6%)	
Right colon	67 (21.0%)	219 (19.0%)		56 (29.0%)	105 (29.5%)	
Rectum	147 (46.1%)	590 (51.1%)		55 (28.5%)	85 (23.9%)	
Family history of cancer, n (%)	40 (12.5%)	116 (10.0%)	0.200	27 (14.0%)	45 (12.6%)	0.655
**Tumor size, n (%)**			0.221			0.281
<2 cm	15 (4.7%)	64 (5.5%)		124 (64.2%)	236 (66.3%)	
2-5 cm	182 (57.1%)	690 (59.7%)		16 (8.3%)	44 (12.4%)	
≥5 cm	122 (38.2%)	401 (34.7%)		53 (27.5%)	76 (21.3%)	
**T stage, n (%)**			<0.001			0.003
T1	9 (2.8%)	99 (8.6%)		5 (2.6%)	17 (4.8%)	
T2	36 (11.3%)	203 (17.6%)		29 (15.0%)	77 (21.6%)	
T3	166 (52.0%)	619 (53.6%)		139 (72.0%)	244 (68.5%)	
T4	108 (33.9%)	234 (20.3%)		20 (10.4%)	18 (5.1%)	
**N stage, n (%)**			<0.001			<0.001
N0	4 (1.3%)	74 (6.4%)		87 (45.1%)	281 (78.9%)	
N1	111 (34.8%)	655 (56.7%)		58 (30.1%)	49 (13.8%)	
N2	121 (37.9%)	254 (22.0%)		48 (24.9%)	24 (6.7%)	
N3	83 (26.0%)	172 (14.9%)		0 (0.0)	2 (0.6%)	
**TNM stage, n (%)**			<0.001			<0.001
Stage I	27 (8.5%)	179 (14.7%)		25 (13.0%)	94 (26.4%)	
Stage II	84 (26.3%)	418 (36.2%)		64 (33.2%)	190 (53.4%)	
Stage III	134 (42.0%)	446 (38.6%)		104 (53.9%)	72 (20.2%)	
Stage IV	74 (23.2%)	121 (10.5%)		0 (0.0)	0 (0.0)	
CEA, ng/mL, IQR*	4.4 (2.2, 12.2)	3.4 (1.8, 8.5)	0.092	3.2 (1.5, 8.3)	2.3 (1.4, 4.1)	0.112
**Chemotherapy, n (%)**			0.106			0.670
Yes	182 (57.1%)	600 (51.9%)		1 (0.5%)	3 (0.8%)	
No	137 (42.9%)	555 (48.1%)		192 (99.5%)	353 (99.2%)	
**Radiotherapy, n (%)**			0.002			0.639
Yes	32 (10.0%)	51 (4.4%)		11 (5.7%)	17 (4.8%)	
No	287 (90.0%)	1104 (95.6%)		182 (46.6%)	339 (95.2%)	
Overall survival months, IQR	25.5 (15.0, 32.1)	31.6 (22.9, 41.5)	<0.001	22.3 (11.1, 33.5)	24.4 (13.0, 38.6)	<0.001
Disease-free survival months, IQR	15.3 (10.0, 22.2)	21.9 (13.5, 31.6)	<0.001	19.9 (10.7, 32.5)	19.3 (11.2, 37.5)	<0.001
Death, n (%)	93 (29.2%)	147 (12.7%)	<0.001	53 (27.5%)	41 (11.5%)	<0.001
Recurrence, n (%)	98 (30.7%)	124 (10.7%)	<0.001	40 (20.7%)	30 (8.4%)	<0.001

*IQR stands for interquantile range.

**Table 2:** Logistic analyses of factors associated with LVI in Wuhan Union cohort

	Univariate analysis		Multivariate analysis	
	OR (95%CI)	*P* value	OR (95%CI)	*P* value
**Age**				
≥60	1.27 (0.99-1.63)	0.057	1.24 (0.95-1.63)	0.119
<60	Ref.	-	Ref.	-
Sex, male	1.04 (0.81-1.34)	0.761		
**Primary site**				
Left colon	1.22 (0.92-1.62)	0.173		
Right colon	1.23 (0.89-1.70)	0.220		
Rectum	Ref.	-		
Family history of cancer	1.28 (0.88-1.89)	0.201		
**Tumor size**				
<2 cm	0.77 (0.42-1.40)	0.392		
2-5 cm	0.87 (0.67-1.13)	0.282		
≥5 cm	Ref.	-		
**T stage**				
T1	Ref.	-	Ref.	-
T2	1.95 (0.90-4.21)	0.089	1.90 (0.84-4.30)	0.124
T3	2.95 (1.46-5.96)	0.003	1.86 (0.86-4.01)	0.113
T4	5.08 (2.47-10.43)	<0.001	2.62 (1.19-5.75)	0.017
**N stage**				
N0	Ref.	-	Ref.	-
N1	3.14 (1.12-8.75)	0.029	3.22 (1.12-9.22)	0.030
N2	8.81 (3.15-24.66)	<0.001	6.11 (2.06-18.15)	0.001
N3	8.93 (3.16-25.25)	<0.001	7.76 (2.63-22.88)	<0.001
**TNM stage**				
Stage I	Ref.	-	Ref.	-
Stage II	1.72 (0.92-3.23)	0.091	1.55 (0.74-3.25)	0.247
Stage III	3.46 (1.90-6.30)	<0.001	1.51 (0.72-3.19)	0.275
Stage IV	14.37 (7.77-26.60)	<0.001	7.53 (3.63-15.63)	<0.001
**Adjuvant chemotherapy**				
Yes	1.23 (0.96-1.58)	0.106		
No	Ref.	-		
**Radiotherapy**				
Yes	2.41 (1.52-3.83)	<0.001	1.91 (1.14-3.21)	0.015
No	Ref.	-	Ref.	-
CEA≥ 5 ng/ml	1.23 (0.96-1.58)	0.110		

**Table 3:** Multivariable Cox analyses of factors associated with OS and DFS in Wuhan Union cohort

	OS		DFS	
	HR (95%CI)	*P* value	HR (95%CI)	*P* value
**Age**				
≥60	1.12 (0.87-1.44)	0.400	-	-
<60	Ref.	-		
Sex, male	1.07 (0.82-1.38)	0.631	-	-
**Primary site**				
Left colon	1.39 (0.95-1.87)	0.060	1.18 (0.87-1.60)	0.297
Right colon	1.40 (1.00-1.98)	0.054	1.33 (0.92-1.93)	0.135
Rectum	Ref.	-	Ref.	-
***Family history of cancer***				
**Tumor size**				
<2 cm	0.87 (0.66-1.14)	0.301	-	-
2-5 cm	1.56 (0.85-2.88)	0.152	-	-
≥5 cm				
**LVI**				
Yes	2.25 (1.70-2.96)	<0.001	2.34 (1.76-3.12)	<0.001
No	Ref.	-	Ref.	-
**T stage**				
T1	Ref.	-	Ref.	-
T2	0.56 (0.27-1.17)	0.123	0.80 (0.62-1.01)	0.064
T3	0.81 (0.44-1.49)	0.493	1.33 (1.06-1.67)	0.015
T4	1.96 (1.07-3.58)	0.031	1.35 (1.05-1.74)	0.021
**N stage**				
N0	Ref.	-	Ref.	-
N1	0.63 (0.34-1.17)	0.606	0.67 (0.35-1.28)	0.227
N2	1.24 (0.64-2.39)	0.991	0.90 (0.46-1.77)	0.758
N3	1.94 (1.01-3.71)	0.047	1.23 (0.63-2.40)	0.539
**TNM stage**				
Stage I	Ref.	-	Ref.	-
Stage II	1.07 (0.48-2.38)	0.863	1.56 (0.56-4.32)	0.396
Stage III	1.27 (0.58-2.81)	0.553	2.28 (0.85-6.15)	0.102
Stage IV	2.66 (1.20-5.91)	0.016	10.49 (3.91-28.17)	<0.001
**Adjuvant chemotherapy**				
Yes	0.56 (0.43-0.73)	<0.001	-	-
No	Ref.	-	-	-
**Radiotherapy**				
Yes	-	-	-	-
No	-	-	-	-
CEA≥ 5 ng/ml	2.10 (1.60-2.76)	<0.001	1.49 (1.13-1.96)	0.005

## Abbreviations

LVI: lymphovascular invasion
CRC: colorectal cancer
PNI: perineural invasion
ROC: receiver operating characteristic
AUC: area under the curve
OS: overall survival
DFS: disease-free survival
MSI: microsatellite instability
